# ADHD GENETIC BURDEN ASSOCIATES WITH COGNITIVE DECLINE AND ALZHEIMER DISEASE MARKERS IN OLDER ADULTS

**DOI:** 10.1093/geroni/igae098.0447

**Published:** 2024-12-31

**Authors:** Thalida Em Arpawong, Jung Ki Kim, Eileen Crimmins

**Affiliations:** University of Southern California, Los Angeles, California, United States; University of Southern California, Los Angeles, California, United States; University of Southern California, Los Angeles, California, United States

## Abstract

Attention deficit hyperactivity disorder (ADHD) is associated with greater risk for Alzheimer disease and related dementias (ADRD). Genetic liability for ADHD, via a well-characterized polygenic score, has shown associations with cognitive decline and AD neurodegenerative markers, but these associations have not been tested in large and diverse samples of older adults. Using data from a population-based Health and Retirement Study, we used linear mixed-effects regression to test associations between an ADHD polygenic score (ADHD-PGS) and longitudinal trajectories of cognitive decline over 12 years among 12,090 White and 3,100 Black adults aged 51 and over. We used linear regression to test the associations between the ADHD-PGS and AD markers, p-tau181 and Abeta 42:40 ratio, among a subset who provided a blood sample (2,361 White and 537 Black older adults). All models were run separately by ethnicity, and controlled for age, sex, APOE4 carrier status, and ancestry principal components. The ADHD-PGS was associated with cognitive decline among White (p< 2e-16) and Black (p=.008) older adults. The ADHD-PGS was associated with p-tau181 (p=0.045) and the Abeta ratio (p=0.039) among White, but not Black older adults (p’s=ns). The ADHD-PGS, which represents the continuum of genetic liability for ADHD-related traits in the population, shows associations with faster cognitive decline over 12 years among a diverse set of older adults. The ADHD-PGS shows statistically significant associations with two neurodegenerative markers among White older adults, but not among a smaller sample of Black older adults, although effect sizes for p-tau were similar across groups.

